# MicroRNA-155 as an inducer of apoptosis and cell differentiation in Acute Myeloid Leukaemia

**DOI:** 10.1186/1476-4598-13-79

**Published:** 2014-04-05

**Authors:** Catalina A Palma, Dima Al Sheikha, Teck Khai Lim, Adam Bryant, Thi Thanh Vu, Vivek Jayaswal, David D F Ma

**Affiliations:** 1 Blood, Stem Cells and Cancer Research, St Vincent’s Centre for Applied Medical Research, St Vincent’s Hospital, Sydney, Australia; 2 School of Mathematics and Statistics, University of Sydney, Sydney, Australia; 3 St Vincent’s Clinical School, Faculty of Medicine, University of New South Wales, New South Wales, Australia

**Keywords:** Acute Myeloid Leukaemia (AML), microRNA, miR-155, Apoptosis, Myeloid differentiation

## Abstract

**Background:**

Acute myeloid leukaemia (AML) is characterised by the halt in maturation of myeloid progenitor cells, combined with uncontrolled proliferation and abnormal survival, leading to the accumulation of immature blasts. In many subtypes of AML the underlying causative genetic insults are not fully described. MicroRNAs are known to be dysregulated during oncogenesis. Overexpression of miR-155 is associated with some cancers, including haematological malignancies, and it has been postulated that miR-155 has an oncogenic role. This study investigated the effects of modulating miR-155 expression in human AML cells, and its mechanism of action.

**Results:**

Analysis of miR-155 expression patterns in AML patients found that Fms-like tyrosine kinase 3 (FLT3)-wildtype AML has the same expression level as normal bone marrow, with increased expression restricted to AML with the FLT3-ITD mutation. Induction of apoptosis by cytarabine arabinoside or myelomonocytic differentiation by 1,23-dihydroxyvitaminD3 in FLT3-wildtype AML cells led to upregulated miR-155 expression. Knockdown of miR-155 by locked nucleic acid antisense oligonucleotides in the FLT3-wildtype AML cells conferred resistance to cytarabine arabinoside induced apoptosis and suppressed the ability of cells to differentiate.

Ectopic expression of miR-155 in FLT3-wildtype AML cells led to a significant gain of myelomonocytic markers (CD11b, CD14 and CD15), increase in apoptosis (AnnexinV binding), decrease in cell growth and clonogenic capacity.

*In silico* target prediction identified a number of putative miR-155 target genes, and the expression changes of key transcription regulators of myeloid differentiation and apoptosis (*MEIS1*, *GF1*, *cMYC*, *JARID2*, *cJUN*, *FOS*, *CTNNB1* and *TRIB2*) were confirmed by PCR. Assessment of expression of apoptosis-related proteins demonstrated a marked increase in cleaved caspase-3 expression confirming activation of the apoptosis cascade.

**Conclusions:**

This study provides evidence for an anti-leukaemic role for miR-155 in human FLT3-wildtype AML, by inducing cell apoptosis and myelomonocytic differentiation, which is in contrast to its previously hypothesized role as an oncogene. This highlights the complexity of gene regulation by microRNAs that may have tumour repressor or oncogenic effects depending on disease context or tissue type.

## Background

Acute myeloid leukaemia (AML) is a heterogeneous disease, which occurs through the accumulation of mutations affecting cell growth and differentiation. The sequence of genetic events leading to disease development remains largely unknown, and microRNAs are postulated to be key mediators of leukaemic transformation, particularly in normal karyotype (NK)-AML [1]. MicroRNAs are short non-coding RNAs that mediate post-transcriptional control of gene expression [2]. The silencing of target mRNA occurs through loading of mature microRNA onto the RNA-induced silencing complex (RISC), which results in mRNA cleavage or translational repression. Through their repressive action, these short 19–25 nucleotide RNA species are important in numerous processes including haematopoietic stem cell maintenance [3] and progenitor self-renewal [4], myeloid differentiation [5], cell cycle and proliferation [6], apoptosis [7] and gene methylation [8].

The *MIR155HG* gene is located at chromosome band 21q21.3, in the exon of a long non-coding RNA transcript from the B cell integration cluster (BIC) [9], and encodes for the microRNA miR-155. This microRNA has emerged as having important roles in haematopoiesis, immunity, inflammation and cancer [10-14], and is the archetypal multifunctional microRNA. In normal host, miR-155 is upregulated in haematopoietic stem cells (HSCs), myeloid progenitor cells, granulocytes, monocytes, macrophages and dendritic cells during maturation and activation, and is also required for normal maturation and function of B and T lymphocytes [12,13].

MiR-155 was first proposed to be oncogenic after it was found to be upregulated in diffuse large B cell lymphoma [9]. Other studies also reported its upregulation in Hodgkin lymphoma [15], chronic lymphoid leukaemia [16], and AML with FLT3-ITD mutations [17]. However miR-155 has also been reported to be downregulated in various haematological malignancies: Burkitt’s lymphoma [18], CML [19], AML with inv(16) [20] and 3q26 cytogenetic abnormalities [21], suggesting that it may play different roles dependent on the type of malignancy. Contradictory roles for microRNAs are not unusual due to their ability to inhibit many target genes. MiR-29 and miR-17-92 cluster, for example, have been shown to have tumour repressor or oncogenic roles depending on disease context or tissue type [22,23].

While the mechanism behind the involvement of miR-155 in B cell lymphoma development has been well studied in murine models [11], the role of miR-155 in AML requires further investigation. The strongest experimental data demonstrating an oncogenic function of miR-155 comes from the overexpression of miR-155 in murine HSCs that led to the development of a myeloproliferative disorder without the development of overt AML. However, these findings have yet to be replicated in a xenograft model using human HSCs, or recapitulated in *in vitro* human HSC cultures [24]. A previous study where miR-155 was overexpressed in human CD34+ cells reported that miR-155 decreased the number and size of myeloid and erythroid colonies [25], but it remains unclear if this was due to a block in differentiation or miR-155 induced growth arrest [26]. Hence, the exact role of miR-155 in human AML remains speculative.

In this study, microRNA expression profiling of normal karyotpe (NK)-AML [27] confirms that miR-155 is overexpressed in patients with FLT3-ITD, but not FLT3-wildtype (WT; which accounts for the majority of NK-AML patients). It demonstrates through overexpression and knockdown *in vitro* studies, that miR-155 has a pro-apoptotic and pro-differentiation role in FLT3-WT AML cells, in contrast to its oncogenic function reported in lymphoma.

## Results

### Expression of miR-155 on normal haematopoietic progenitors and AML cells

**Figure 1:**
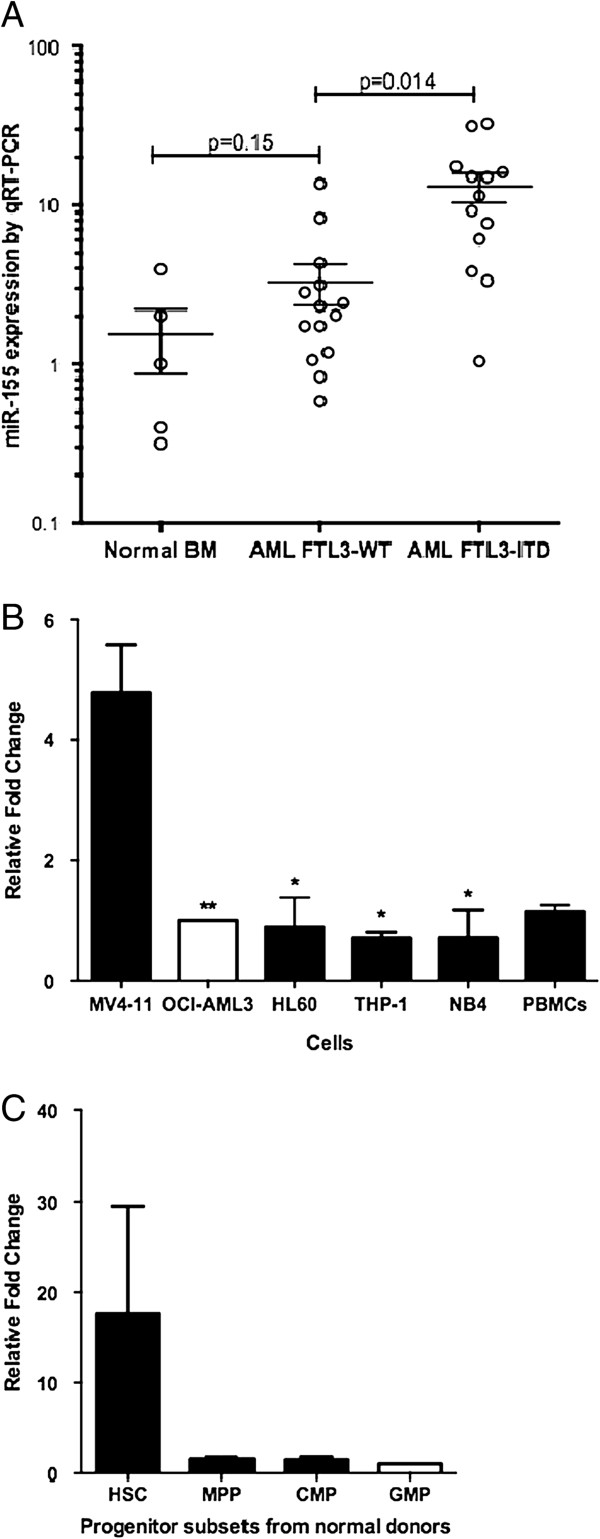
**miR-155 expression in AML samples and normal haematopoietic cells. (A)** miR-155 expression levels by qRT-PCR in normal bone marrow (5 samples) and AML samples with FLT3-ITD (13 samples) or wildtype (14 samples), values are normalized to the mean of the normal bone marrow samples **(B)** miR-155 expression in selected AML cell lines and PBMCs, presented as mean fold change + SEM relative to OCI-AML3 (baseline set at 1, white bar). Unpaired Two Tailed T-Test compared to expression levels in MV4-11 cells, *p < 0.05, **p < 0.001. n = 3-5 **(C)** miR-155 expression levels of haematopoietic progenitor subsets isolated from four normal mobilized peripheral blood donors, presented as mean + SEM relative to GMP. No statistical difference detected between groups using Paired t-test. HSC- haematopoietic stem cell; MPP- multipotent progenitor; CMP- common myeloid progenitor; GMP- granulocyte monocyte progenitor.

Analysis of microRNA expression by qRT-PCR in NK-AML samples compared to normal bone marrow found miR-155 to be significantly overexpressed in patients exhibiting the FLT3-ITD mutation (4.06- fold increase, p = 0.014), but not FLT3-WT (2.14-fold, p = 0.15) (Figure 1A). This pattern was recapitulated by AML cell lines (Figure 1B), where the highest expression was harboured by MV4-11 cell line (4.78 ±0.80, mean fold change relative to OCI-AML3± SEM) which expresses the FLT3-ITD mutation [28]. The majority of cell lines tested exhibited similar miR-155 expression patterns to normal human PBMCs (1.15±0.11, Figure 1B).

To compare miR-155 expression between AML and normal haematopoietic cells, microRNA expression was measured in progenitors isolated from human mobilized peripheral blood. Progenitors were sorted into human HSCs (Lin- CD34+ CD38- CD90+ CD45RA-), MPPs (multipotent progenitors; Lin-CD34+ CD38- CD90- CD45RA-), CMPs (common myeloid progenitors; Lin- CD34+ CD38+ CD123+ CD45RA-) and GMPs (granulocyte-macrophage progenitors (GMPs; Lin- CD34+ CD38+ CD123+ CD45RA+) as described previously [29,30]. Analysis of miR-155 expression in normal haematopoietic progenitors revealed a trend towards higher expression, similar to that found in FLT3-ITD AML, in purified HSCs (17.64±11.88, presented as mean fold change relative to GMP± SEM, Figure 1C). Expression of miR-155 then appeared to decrease as cells progressed through MPP (1.517±0.28), CMP (1.45±0.32) and GMP stages (Figure 1C).

### Bioinformatic prediction of miR-155 targets reveals enrichment for transcription factors that regulate myeloid cell growth and differentiation

To discover the potential target genes of miR-155, three bioinformatics databases (TargetScan, microRNA.org and MicroCosm) were utilised to predict miR-155 3’UTR recognition elements across the transcriptome of protein coding genes as described by Bryant et al. [31]. 585 genes were common to at least two databases and 75 genes were common to three databases (listed in Additional file 1: Table S3). Gene ontology (GO) analysis of the permissive gene target list using DAVID Functional Annotation tool found an enrichment for genes related to Transcription, Regulation of Transcription, Myeloid Cell Differentiation, Apoptosis and Programmed Cell Death amongst others (Additional file 2: Table S1). From these predicted targets, genes were selected that may have a role in myeloid growth, myeloid differentiation and leukaemia development as determined by literature searches and GO terms.

**Figure 2:**
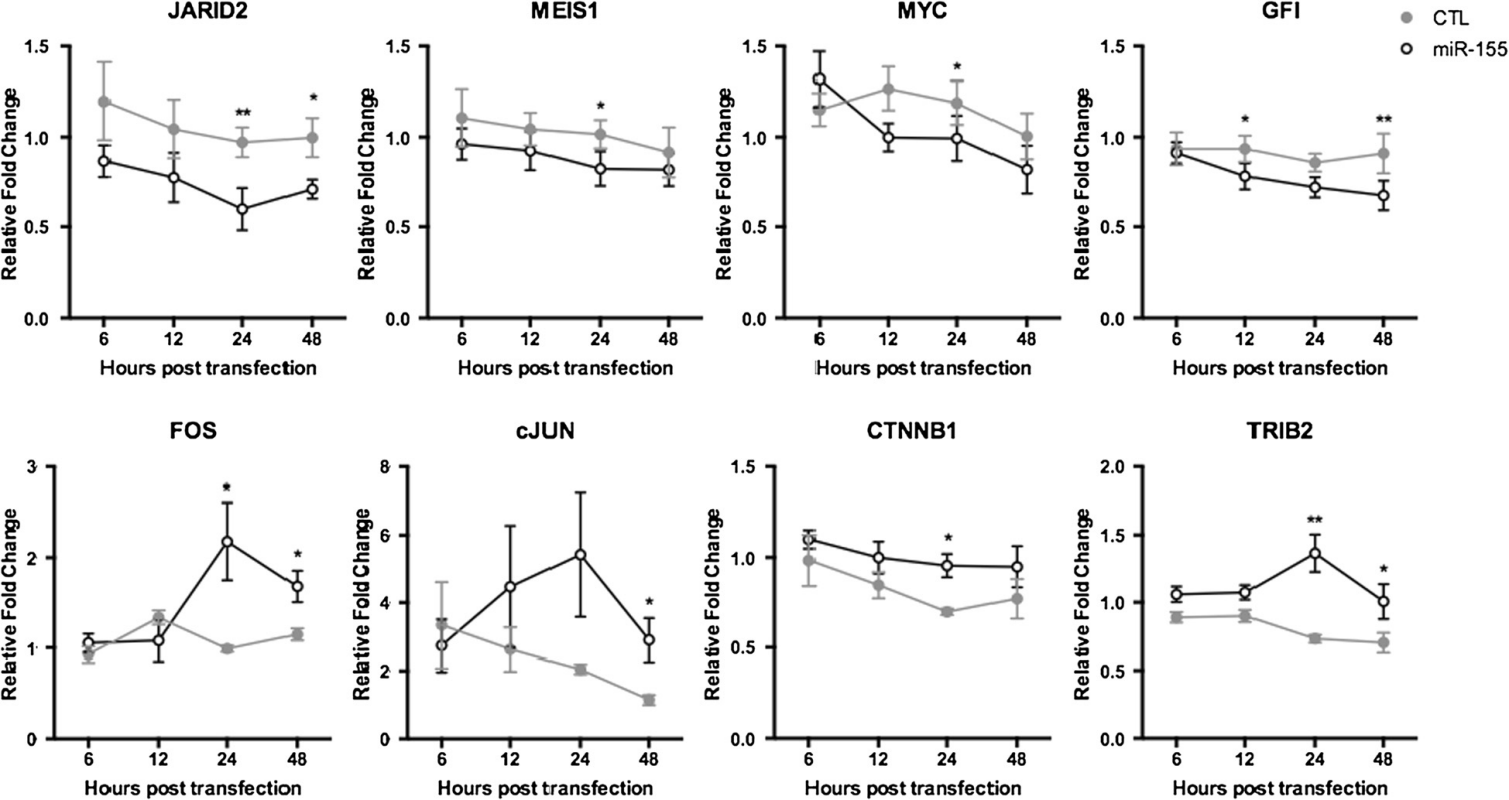
**Effects of miR-155 transfection on the expression of selected putative target genes over 48 hours.** Assessed by qRT-PCR at 6, 12, 24 and 48 hours after transfection of OCI-AML3 cells with Scrambled control (CTL, grey line) or miR-155 (black line). Results are depicted as mean fold change in mRNA expression + SEM relative to untreated control cells with baseline set to 1.0 (not shown). β-actin was used as the reference gene. Statistical significance was assessed using a Paired Two Tailed T-Test. *p < 0.05, **p < 0.01; n = 5-8.

The expression levels of 20 genes, putative miR-155 targets as well as key mediators of apoptosis and myeloid growth were evaluated (Additional file 3: Table S2). The mRNA levels of target and pathway related genes were examined in OCI-AML3 cell line (FLT3-WT AML) transfected with miR-155 or scrambled control for 6, 12, 24 and 48 hours (Figure 2). In OCI-AML3 cells, miR-155 was found to downregulate the oncogenic genes: *MEIS1* (decreased by 19.5 ± 5.82% (p < 0.05) at 24 hours), *GFI1* (decreased by 24.28 ± 4.56% (p < 0.01) at 48 hours) and *MYC* (decreased by 14.12 ± 6.58% (p < 0.05) at 24 hours). *JARID2,* part of the polycomb repressive complex, was downregulated at 24 hours (39.37 ± 8.29%, p < 0.01) and 48 hours (27.65 ± 3.84%, p < 0.05), which itself has many downstream gene targets. Interestingly, an increase in the validated miR-155 target genes *c-JUN*[32] and *FOS*[33], which form the AP-1 transcriptional complex, was detected. *c-JUN* increased at 48 hours (136.8 ± 31.62%, p < 0.05) while *FOS* expression increased at both 24 and 48 hours (121.8 ± 43.46% and 46.03 ± 11.8% respectively, p < 0.05), suggesting that in this system miR-155 is not negatively targeting these genes. The presence of an intermediary gene may explain this positive regulation. *CTNNB1,* which codes for β-catenin, was also significantly increased at 24 hours (36.99 ± 8.56%, p < 0.05). *TRIB2* a coactivator of the HOXA9/MEIS1 oncogenic pathway, was increased at 24 hours (89.46 ± 21.60%, p < 0.01) and 48 hours (44.14 ± 10.70%, p < 0.05). There were no detectable changes in gene expression of *HOXA9, PBX1, FOXO3, CEBP*α*, CEBP*β*, CCND1, JAK2, ARNTL, EPAS1, HIF1*α*, CXCR4* or *HDAC4* at any time point (data not shown).

### Altered miR-155 expression results in changes in FLT3-WT AML cell differentiation

**Figure 3:**
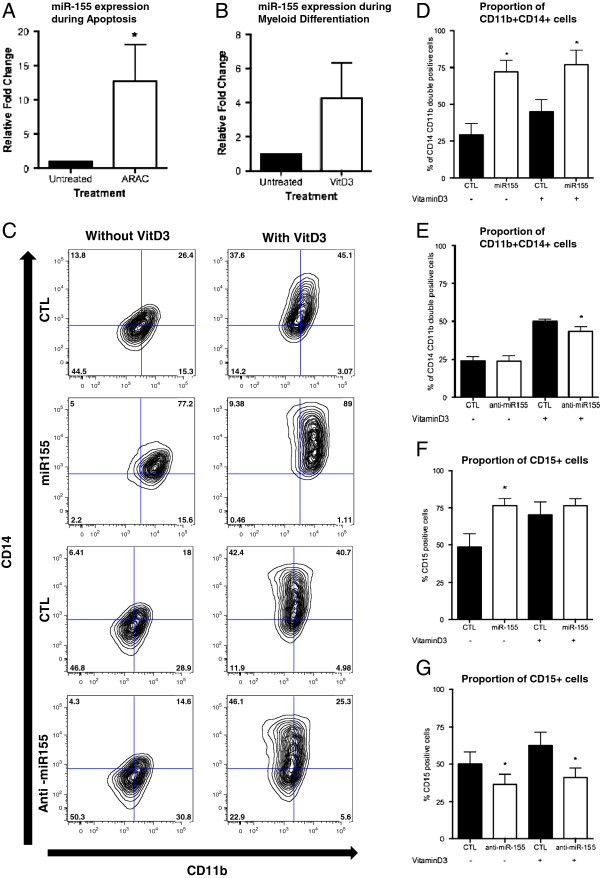
**miR-155 expression levels affect myeloid differentiation of AML cells.** Expression of miR-155 48 hours after **(A)** ARAC induced apoptosis of OCI-AML3 cells or **(B)** VitD3 induced monocytic differentiation of OCI-AML3 cells. Data is presented as mean fold change expression of miR-155 + SEM relative to Untreated control; RNU6b was used as the reference gene, n = 3. VitD3 was used to induce myeloid differentiation in OCI-AML cells transfected with miR-155, anti-miR-155 or their respective controls **(C)** flow cytometry plots of phenotype analysis using CD11b and CD14 **(D)** miR-155 overexpression increased the percentage of CD11b + CD14+ cells at 48 hours **(E)** percentage expression of CD14 + CD11b + cells transfected with anti-miR155 and exposed to VitD3 (+) or PBS (-) for 48 hours **(F)** miR-155 overexpression increased the percentage of cells expressing CD15 at 48 hours without VitD3 stimulation **(G)** miR-155 inhibition decreased the percentage of cells expressing CD15. CTL- scrambled control; ARAC- cytarabine arabinoside; VitD3- 1,23-dihydroxyvitaminD3. Statistical significance determined using Paired Two Tailed *t*-Test, *p < 0.05; n = 4.

To determine how miR-155 expression affects cell function, the change in miR-155 expression in differentiation and apoptosis processes were assessed. It has been previously reported that during macrophage differentiation and activation, miR-155 expression increased 60-fold [10], suggesting that an increase in miR-155 expression is involved in myeloid cell maturation. To determine the typical miR-155 expression pattern during monocytic differentiation and apoptosis, MV4-11 and OCI-AML3 cells were treated with 1,23-dihydroxyvitaminD3 (VitD3, a known inducer of monocytic phenotype [27]) or cytarabine arabinoside (ARAC). OCI-AML3 cells exhibited a significant increase in miR-155 expression after induction of apoptosis (3.85 fold, p = 0.019, Figure 3A) and a trend towards an increase during monocytic differentiation (3.04 fold, Figure 3B). However the FTL3-ITD mutated MV4-11 cell line exhibited no significant change of miR-155 expression after induction of apoptosis (Additional file 4: Figure S1A), and a trend towards decrease in miR-155 expression during induction of monocytic differentiation at 48 hours after VitD3 treatment (p = 0.1513, Additional file 4: Figure S1B).

To determine if modulating miR-155 expression could impact monocytic differentiation of FLT3-WT AML cells, OCI-AML3 cells were transfected with synthetic miR-155 or anti-miR-155 LNA (and matched scrambled controls), in the presence or absence of VitD3. Analysis of OCI-AML3 phenotype using CD11b (myeloid) and CD14 (monocyte) markers (Figure 3C), found that ectopic expression miR-155 induced a significant 2.48-fold increase in cells positive for both CD11b and CD14 after 48 hours, which was not further increased by VitD3 treatment (1.71-fold increase compared to Scrambled control, Figure 3D). miR-155 overexpression also induced an increase in CD15 (granulocyte marker) positive cells at 48 hours (Figure 3F).

Conversely, miR-155 inhibition by anti-miR-155 in OCI-AML3 cells decreased the monocytic differentiation potential of the cells upon VitD3 treatment by 13.42% (p = 0.0324, Figure 3E), but had no significant effect on the phenotype of unstimulated cells. MiR-155 knockdown also decreased the proportion of CD15 positive cells and inhibited the gain of CD15 upon VitD3 treatment (Figure 3G). Transfection of FLT3–ITD mutated MV4-11 cells with anti-miR-155 failed to demonstrate significant differences in proportion of CD11b and CD14 positive cells, even after treatment with VitD3 (p = 0.2718 without VitD3 induction, and p = 0.1761 with VitD3 induction, Additional file 5: Figure S2A).

### miR-155 expression can regulate AML cell survival

**Figure 4:**
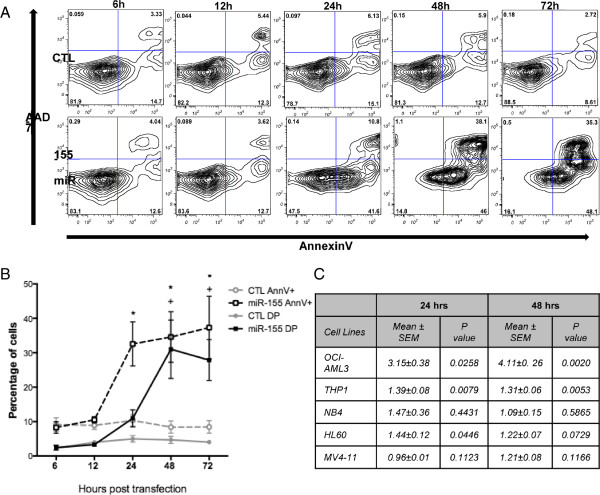
**Induction of apoptosis of OCI-AML3 cells after miR-155 transfection. (A)** Gating strategy for flow cytometry analysis. Cells undergoing early apoptosis are stained for AnnexinV, cells undergoing late apoptosis are stained for both AnnexinV and 7-AAD **(B)** increase in AnnexinV positive cells (dashed line) or Double Positive cells for AnnexinV/7-AAD (solid line) post transfection with miR-155 (black line) compared to CTL (grey line) **(C)** summary of fold-change of cells (OCI-AML3, THP1, NB4, HL60, MV4-11) binding AnnexinV overexpressing miR-155 normalised to CTL at 24 and 48 hours post transfection. AnnV + AnnexinV positive cells; DP- double positive cells, CTL- Scrambled control. Statistical significance determined using Paired Two Tailed *t*-Test, *p < 0.05 of AnnV + cells; +p < 0.05 of DP cells, n = 4-7.

To assess the effects of miR-155 expression in AML cell survival, the human cell lines OCI-AML3 (AML- M4), THP1 (AML- M5), NB4 (AML- M3), HL60 (AML- M2) and MV4-11 (AML- M4) were used. Cells were transfected with synthetic miR-155 or scrambled control and assayed with annexinV, a marker of early apoptosis, and 7-AAD, a marker of late apoptosis, at varying time points (Figure 4). In OCI-AML3 cells, a strong increase in annexinV binding was detected at 24, 48 and 72 hours after transfection with miR-155 compared to control, followed by an increase in annexinV/7AAD double positive cells at 48 and 72 hours (Figure 4A, B). This latency in the gain of 7AAD staining reflects the passage of cell through the early apoptotic stages, to cell necrosis. The THP1 cell line also demonstrated significantly increased apoptosis with miR-155 overexpression, with 1.39±0.08 and 1.31±0.06 fold increase in annexinV positive cells compared to scrambled control at 24 and 48 hours respectively. HL60 cells demonstrate a significant increase in annexinV binding at 24 hours, which was attenuated at 48 hours. While the NB4 cell line demonstrated a trend towards an increase at 24 hours (1.47±0.36), but this was not significant. The survival of MV4-11 cells was not significantly affected by an increase in miR-155 levels (Figure 4C).

**Figure 5:**
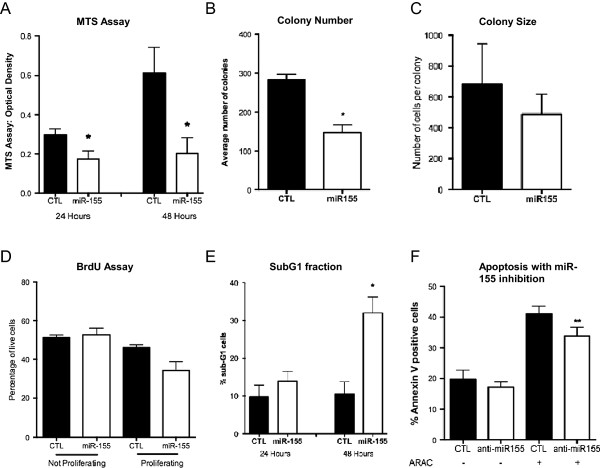
**Growth perturbations in AML cells with altered miR-155 expression levels. (A)** Transfection of OCI-AML3 cells with miR-155 resulted in a reduction of cell growth at 24 h and 48 h as determined by MTS assay **(B)** Assessment of colony forming potential found a decrease in mean colony number and **(C)** a trend towards reduced colony size (presented as mean + SEM cells per colony) **(D)** Proliferation through BrdU incorporation did not demonstrate a significant change **(E)** Cell cycle analysis demonstrated an increase in the proportion of cells in the SubG1 fraction at 48 h post miR-155 overexpression. CTL, Scrambled Control **(F)** Transfection of OCI-AML3 cells with anti-miR155 LNA resulted in a significant decrease in the proportion of cells undergoing apoptosis (AnnexinV+) when treated with ARAC, but not when untreated. ARAC-cytarabine arabinoside; LNA-locked nucleic acid; CTL, Anti-Scrambled LNA. Statistical significance determined using Paired Two Tailed *t*-Test, *p < 0.05 ** p < 0.001; n = 3-4.

The pro-apoptotic role of miR-155 was further supported by MTS assay of OCI-AML3 cells overexpressing miR-155 or scrambled control, which demonstrated a significant reduction in cell number at 24 and 48 hours (41.7% (p = 0.017) and 66.8% (p = 0.048) respectively, Figure 5A).

Assessment of the clonogenic potential of cells overexpressing miR-155 or control by growth in semi-solid media (Figure 5B), demonstrated a reduction in colony formation by miR-155 overexpressing cells (147±19 colonies per 35 mm dish versus 282±14 colonies of control cells, p = 0.027), with a concurrent trend towards smaller colony size (487±130 vs 681±262 cells per colony, Figure 5C). Cell cycle analysis by PI incorporation (not shown) or BrdU (Figure 5D) uptake did not demonstrate a significant change in the proportion of cells in G1, S or G2/M phases. Both assays however did detect a significant increase in the number of cells in the Sub-G1 fraction (3.04-fold increase at 48 hours, p = 0.0025, Figure 5E), further supporting an induction of cell death by miR-155 overexpression.

To support this finding, miR-155 expression was inhibited in OCI-AML3 and MV4-11 cells using anti-miR-155 LNA or Scrambled LNA control, and apoptosis levels were assessed by annexinV/7AAD binding assay. OCI-AML3 cells exhibited significantly less apoptosis upon treatment with ARAC than control cells (33.82±2.72% annexinV binding in anti-miR-155 cells versus 40.93±2.58% in control cells, p = 0.0007, Figure 5F), suggesting that inhibiting miR-155 levels protects cells from cell death inducers like ARAC. Apoptosis levels of untreated OCI-AML3 (Figure 5F) or MV4-11 (Additional file 5: Figure S2B) cells were not changed by miR-155 knockdown.

### Increased expression of miR-155 activates the apoptosis cascade in AML cells

To identify the apoptotic mechanisms responsible for the increase in cell death caused by miR-155 overexpression, the levels of apoptotic proteins were profiled by the R&D Systems Proteome Profiler Apoptosis Array in OCI-AML3 cells transfected with synthetic miR-155 or Scrambled control, in three independent repeats. The Antibody Array demonstrated trends towards increased expression of pro-apoptotic proteins (p27/kip1, BAD, FAS, Procaspase-3, Cleaved Caspase 3, phosphorylated p53, OMI/HTRA2) and anti-apoptotic proteins (XIAP, BCL-X, HSP32, Clusterin) with at least a 1.5-fold increase in expression in miR-155 overexpressing cells compared to control (Table 1).

**Table 1:** miR-155 overexpression induces changes in levels of apoptotic proteins

**Protein**	**Mean**	**SEM**
Bad	1.5	0.52
Bax	1.39	0.27
Bcl-2	1.13	0.2
Bcl-x	1.83	0.29
Catalase	1.24	0.31
clAP-1	1.47	0.35
clAP-2	1.41	0.27
Claspin	1.3	0.13
Cleaved caspase 3	2.5	0.21
Clusterin	1.8	0.24
Cytochrome C	1.09	0.04
FADD	1.48	0.19
FAS/TNFRSF6	2.32	0.94
HIF-1a	1.46	0.27
HO-1/HMOX1/HSP32	2.14	0.63
HO-2/HMOX2	1.39	0.03
HSP27	1.33	0.07
HSP60	1.4	0.03
HSP70	1.22	0.17
HTRA2/Omi	1.54	0.33
p21/CIP1/CDNK1A	1.06	0.32
p27/Kip1	1.89	0.81
phospho-p53 (S15)	1.33	0.42
phospho-p53 (S392)	1.6	0.68
phospho-p53 (S46)	1.8	0.8
phospho-p53 (S635)	1.5	0.42
PON2	0.88	0.24
Pro-caspase 3	1.72	0.61
SMAC, Diablo	1.37	0.1
Survivin	1.24	0.22
TNFRI/TNFRSF1A	1.25	0.22
TRAIL R1/DR4	0.76	0.13
TRAIL R1/DR5	0.8	0.06
XIAP	2	0.44

Apoptosis array results indicating mean fold-change (with SEM, n = 3) of apoptosis related proteins between Scramble control and miR-155 transfected cells.

**Figure 6:**
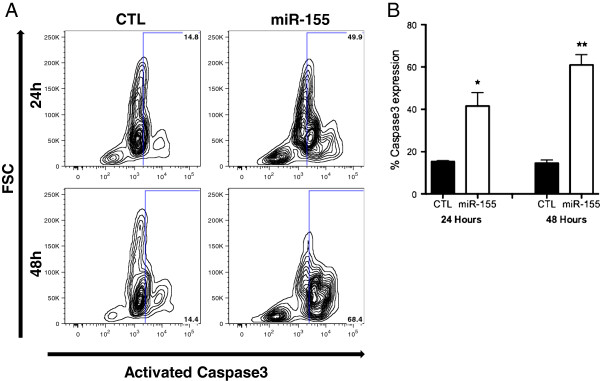
**Effect of miR-155 overexpression on the expression of Cleaved Caspase-3.** Cleaved caspase-3 expression was confirmed to be upregulated in miR-155 transfected cells by a flow cytometry based assay **(A)** flow plots of activated caspase-3 in miR-155 overexpressing or CTL cells at 24 h and 48 h **(B)** significant increase in the percentage of cells expressing activated caspase-3. Statistical significance determined using Paired Two Tailed *t*-Test, *p < 0.05, **p < 0.01; n = 4.

We confirmed that apoptosis was occurring through caspase-3 activation, by probing for cleaved caspase-3 expression by flow cytometry (Figure 6). Results show that caspase-3 activation is increased 2.73-fold (p < 0.05) and 4.19-fold (p < 0.01) at 24 and 48 hours (respectively) after miR-155 transfection compared to scrambled control, demonstrating that high miR-155 expression induces apoptosis in these cells through caspase activation.

## Discussion

MiR-155 has been linked to the development of haematopoietic malignancies, however it remains a critical part of normal development, with its expression essential for haematopoietic cell maturation, immune cell differentiation and function. It also has established roles in maintaining homeostatic regulatory loops in the immune system. Within the context of AML development, miR-155 has been cited as oncogenic, with increased expression in AML with the FLT3-ITD mutation, and recently, its correlation with poor survival in subsets of older patients [34]. Our study demonstrated pro-apoptotic and pro-differentiation functions for miR-155, hence an anti-leukaemia role in AML, highlighting the complexity of microRNA based regulation and challenging our current understanding of miR-155 functions in myeloid development and malignancy.

In this study we showed that AML patients with FLT3-ITD mutation express a higher level of miR-155 than FLT3-WT patients or normal controls. This supports the finding of Garzon et al. [35] who reported a ~5-fold increase in FLT3-ITD AML patients. They further demonstrated that miR-155 expression is independent of FLT3-ITD signal. In our study, AML cell lines recapitulated this expression pattern, with MV4-11 cells expressing the highest miR-155 level compared to FLT3-WT cell lines (NB4, THP1, OCI-AML and HL60). FLT3-WT cell lines expressed similar levels of miR-155 to normal human PBMCs.

Gene target prediction was used as a preliminary indicator of miR-155 function. Bioinformatic prediction followed by GO analysis using the DAVID Annotation Tool yielded a set of target genes which focused heavily on transcriptional modulators, as well as myeloid differentiation genes and apoptosis regulators (Additional file 2: Table S1). qRT-PCR analysis for a set of 20 genes provided evidence of potential functions for miR-155 in AML cells. We observed the upregulation of miR-155 during induced apoptosis and monocytic differentiation in FLT3-WT AML, suggesting its involvement in these processes.

Our experimental data support a role for miR-155 in the AML maturation block, with its overexpression inducing the expression of CD11b, CD14 and CD15 surface markers in over 70% of cells. This phenotype is consistent with cells released from differentiation block [36]. Further, knockdown of miR-155 in these cells decreased their ability to differentiate upon stimulation with VitD3. This ability of miR-155 to induce maturation is consistent with its postulated role in myeloid cell lineage commitment from HSCs and myeloid progenitors. MiR-155 expression is required for the differentiation of myeloid cells into functional macrophages [10,37] and dendritic cells [38], and myeloid differentiation of murine HSCs in colony forming assays [25]. This pro-differentiation function of miR-155 is supported by its involvement in key genetic networks in haematopoietic differentiation. *PU.1*, a master haematopoietic transcription factor, a target of miR-155, is necessary for myeloid differentiation of HSCs [39], while *AP-1* and *NF-*κ*B* transcription factors are postulated to regulate miR-155 expression in macrophages [10].

In this study, bioinformatic prediction combined with pathway analysis and validation by qRT-PCR revealed that miR-155 expression positively correlates with the expression of the AP-1 genes *c-JUN* and *FOS,* which are known to induce myeloid differentiation [40]. They were also detected upon the release of the AML maturation block in this study. MiR -155 expression was found to downregulate the oncogenic *MEIS1* and *GFI1* genes, which work in synergy with HOXA9 to accelerate AML transformation [41]. Normally GFI1 functions as a key regulator of HSC and neutrophil development, and its upregulation is leukaemogenic [42]. *MEIS1* is oncogenic in murine bone marrow through the activation of *TRIB2*, which in turn inhibits *C/EBP*α, halting myeloid differentiation [43]. We detected no change in *C/EBP*α expression, suggesting that the induction of myeloid maturation is occurring through an alternate pathway. *JARID2* is a confirmed target of miR-155 by luciferase assay [44] and is downregulated by miR-155 overexpression in this study. *JARID2* is a co-activator and recruiter of the Polycomb repressive complex 2 (PRC2) responsible for the repression of a wide range of target genes [45].

Together with an induction of myeloid maturation, the overexpression of miR-155 was found to induce apoptosis in OCI-AML3, THP1 and HL60 cell lines as detected by AnnexinV/7AAD assay. The strong apoptotic response seen in the OCI-AML3 cells were confirmed by MTS assay and the increased sub-G1 cell population. This finding supports previous observations of increased sub-G1 population and partial differentiation of THP1 cells [26], and induction of apoptosis in dendritic cells [38] upon miR-155 overexpression. Importantly, our study also demonstrated that knockdown of miR-155 conferred protection from chemotherapy insult. In tumor clonogenic assays miR-155 overexpression resulted in decreased colony number and size. This supports the study by Georgantas et al., where miR-155 overexpression in normal human CD34+ cells generated fewer and smaller myeloid and erythroid colonies [25].

**Figure 7:**
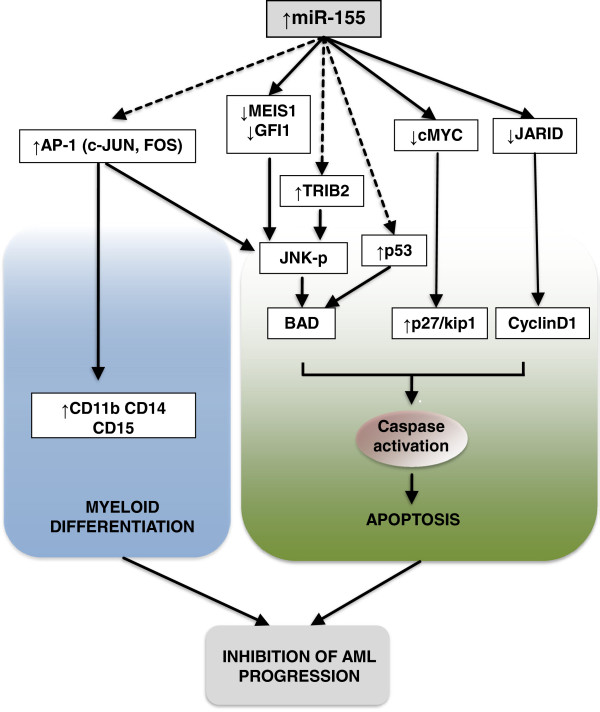
**Potential regulatory mechanisms of miR-155 in AML.** Model of miR-155-mediated gene regulation in FLT3-WT AML, where its expression acts to induce myeloid differentiation and caspase-3 dependent apoptosis. In AML cells miR-155 overexpression was found to decrease the expression of *cMYC* and *MEIS1/GFI1*, potentially leading to the phosphorylation of JNK and/or the activation of p27/kip1 and activation of the apoptosis cascade. The increase in *AP-1* and *TRIB2* genes may be caused by an intermediary, such as *JARID2* inhibition, and may have functions in regulating AML cell differentiation and apoptosis.

We found overexpression of miR-155 led to increase in *cJUN, FOS* and *TRIB2,* and decrease in *MEIS1*, *GFI1, cMYC* and *JARID2*. Induction of AP-1 transcription factors have been reported to result in increased phosphorylation activity of JNK and activation of caspase-3 dependent apoptosis in AML [46,47]. Decrease in *MEIS1*, *GFI1* and *cMYC*, has been shown to inhibit tumour cell growth, and an increase in *TRIB2* may be pro-apoptotic [48]. Our study demonstrates that miR-155 induces apoptosis through a caspase-3 mediated mechanism. This increase in cleaved caspase-3 was accompanied by a trend towards increased levels of pro-apoptotic p27/kip1, BAD, procaspase-3, phosphorylated p53, FAS and HSP32 as well as increased levels of the anti-apoptotic XIAP, BCL-XL and CLUSTERIN. We postulate that miR-155 induces apoptosis via a combination of p53 activation, p27/kip induction via downregulation of *cMYC*[49], phosphorylation of JNK by increased AP-1 or *TRIB2* expression, or regulation of cyclinD1 by *JARID2* downregulation [50] (Figure 7).

MiR-155 has been postulated as oncogenic [9,11,24], however our findings show induction of apoptosis and differentiation upon miR-155 overexpression in AML cells. There are a number of plausible explanations for this dual effect. Firstly, miR-155 may have different targets in different subtypes of AML as reported for microRNA-17-92 in different B-cell lymphoma subtypes [51]. Secondly, miR-155 could target both oncogenes and tumour suppressors to varying degrees within the same cells as reported for miR-196b [52], and might favour one over the other in particular subtypes of AML. Thirdly, miR-155 may have different functions depending on its expression level. At functionally high but transient levels of miR-155, such as during the immune response, cells are physiologically normal, however, at prolonged intermediately high levels miR-155 appears to be oncogenic [14]. This could be due to escaping of feedback inhibitory loops, targeting of different transcripts or dose-dependent effects that different miR-155 levels may have on the same transcripts.

We acknowledge that the strength of the conclusions of this study are hampered as they are based on *in vitro* experiments and clinical observational studies. The functional role of miR-155 should be further studied using animal models and primary AML samples to elucidate the role of miR-155 in the distinct molecular networks within this heterogeneous disease. Comprehensive studies of miR-155 gene targets (using HITS-CLIP for example) in AML are required. However, our study adds to an increasing pool of evidence [19,26] that miR-155 has an anti-tumour function and is not only pro-tumourigenic.

In a recent study Marcucci et al. [34] reported that higher miR-155 expression levels had a negative impact on the outcome of AML in patients, most markedly in younger patients. Although this is a significant study and provides an important novel outcome marker for AML in younger patients, it does not help clarify the biological actions of miR-155. In support of our findings, microarray transcriptome analysis of AML patients found the strong positive correlations between miR-155 expression and pro-apoptosis genes (GO:0006915-Apoptosis) and haematopoietic pro-differentiation genes, as well as anti-apoptosis genes, suggesting that miR-155 is inducing expression of anti-tumour and pro-tumour genes in the same cohort.

In lung cancer, contrary the report by Marcucci et al., low miR-155 expression correlates with aggressive disease with increased occurrence of metastases [53], similarly in breast cancer where a lack of miR-155 expression links with disease progression [54]. Our analysis on publically available clinical databases [55-57] demonstrated that high miR-155 expression can correlate with significantly longer OS in patients with prostate or high-risk breast cancer (Additional file 6: Figure S3). In summary, these clinical studies lend support to our finding that miR-155 can have ant-tumour functions.

## Conclusions

This study provides novel evidence for an anti-leukaemia function of miR-155 in AML by inducing cell apoptosis through caspase-3 activation, and releasing the myeloid differentiation block. Overexpression of miR-155 was found to alter the expression of transcription factors and chromatin modifiers that regulate myeloid cell survival and differentiation, and upregulate mediators of the apoptotic cascade. The precise downstream pathways targeted by miR-155 remain to be elucidated. Our study provides evidence that the gene regulation by miR-155 is complex, as it targets both tumour suppressors and oncogenes, and its function in a specific malignancy is likely subject to disease context and cell type.

## Materials and methods

### Cell lines and patient samples

This study was reviewed and approved by The St Vincent's Hospital Human Ethics Committee (HREC 06/064). Cell lines OCI-AML3 and HL60 cells purchased from the DSMZ cell-line bank (Braunschweig, Germany), and MV4-11, NB4 and THP1 cells were purchased from ATCC (Virginia, USA). Isolation of haematopoietic progenitors was performed on donated mobilized peripheral blood product. Diagnostic bone marrow samples of 27 NK-AML cases with leukemic blast counts of at least 50% were obtained after informed consent from the tissue banks of our institute and the Australian Leukaemia and Lymphoma Group Tissue Bank. Five normal bone marrow samples were obtained from our institute’s tissue bank for comparison. Microarray was performed as described in [31]. To determine FLT3 mutational status, the insertional hotspot was RT-PCR amplified from complementary DNA using previously described primers [31] and directly sequenced on the 3100XL from ABI 3100XL Genetic Analyser. 13/27 (48.1%) samples harboured a mutation in the FLT3 gene.

To confirm miR-155 expression in primary samples and comparison of expression in cell lines, 1 μg of total RNA was used for cDNA synthesis reaction using the NCode microRNA first strand synthesis kit (Invitrogen, USA). Quantitative real time polymerase chain reaction (qRT-PCR) was performed using the Platinum SYBR Green qPCR SuperMix-UDG (Life Technologies, USA) and the Rotorgene RG-3000 thermocycler (Qiagen, Hilden, Germany). RNU6b and RNU43 were used as the reference genes (primer sequences in Additional file 7: Table S4). For functional cell line work, microRNA expression was quantified by the TaqMan miRNA assay (Applied Biosystems) as per manufacturer’s instructions. All qPCR reactions were performed in duplicate and RNU6b was used as the reference gene. MicroRNA expression data are expressed as mean fold change in gene expression (+SEM) relative to control. Two-tailed T-tests were performed on ΔCt values to determine significant differences (defined as p < 0.05) between two groups.

### Fluorescence-activated cell sorting (FACS) of human haematopoietic progenitors

Mobilised peripheral blood samples were thawed, washed in PBS and incubated at 37°C with PBS/1% FBS/1 mM EDTA (FACS Buffer) for 30 mins and non-adherent cells collected to remove monocytes. Lineage-negative cells were collected by using EasySep® Human Progenitor Cell Enrichment Kit (STEMCELL Technologies, Victoria, Australia). Cells were incubated with EasySep® Enrichment Cocktail followed by EasySep® Magnetic Nanoparticles. Cells were then incubated for 10 mins in the EasySep® magnet, where lineage-negative cells were magnetically separated. Lineage-negative cells were stained with the following fluorophore-conjugated antibodies: Lineage-FITC, CD34-phycoerythrin (PE)Cy7, CD38-PerCP-Cy5.5, CD45RA-PacificBlue, CD90-APC, CD123-PE (BD Biosciences, San Jose, CA, USA). Cells were FACS sorted and analyzed using BD Influx Cell Sorter (BD Biosciences, San Jose, CA, USA). Cells were collected in sterile FACS buffer, and stored at -80°C in QIAzol (Qiagen, Valencia, CA, USA) until RNA extraction.

### miR-155 target prediction and pathway analysis

Three bioinformatic databases were used for the *in silico* prediction of miR-155 targets: TargetScan [58], microRNA.org [59,60] and MicroCosm [61]. Predicted target genes were obtained that were common to at least 2 of the 3 databases or 3 of the 3 databases (accessed on 27/7/2012). Selected target genes were chosen for qRT-PCR analysis based on their putative involvement in AML development. The DAVID Functional Annotation Tool (http://david.abcc.ncifcrf.gov/summary.jsp) was used to classify the genes into ontologically-related terms and then examine for enriched terms [62].

RNA was extracted from cell samples using TRizol (Life Technologies) according to the manufacturers instructions. 1 μg of total RNA was used in the cDNA synthesis reaction using SuperScriptIII reverse transcription kit (Life Technologies). qRT-PCR was performed using the Platinum SYBR Green qPCR SuperMix-UDG on a Rotor-Gene 3000. Data were analysed using the ΔΔCT method. β-Actin was found to be stably expressed in these experiments, and was thus used as the reference gene. Primers are listed in Additional file 7: Table S4. Gene expression data were expressed as mean fold change in gene expression (+SEM) relative to control. *t*-tests were performed to determine significant differences defined as p < 0.05.

### miR-155 overexpression and antisense repression

Cell lines were cultured in RPMI or α-MEM solution with 10% fetal bovine serum (GIBCO). Cells were transfected with 50 nM pre-miR-155 (Ambion) or anti-miR155 miRCURY LNA™ microRNA knockdown probes (Exiqon, Denmark) or appropriate scrambled control using Lipofectamine 2000 and Opti-MEM (both from LifeTechnologies, USA) in antibiotic-free media. Lipofectamine based transfection was found to cause on average 3.6% cell death. Cells were maintained in a humidified incubator at 37°C in 5% CO_2_. miR-155 overexpression and knockdown was confirmed by TaqMan qRT-PCR as per manufacturer’s instructions (Applied Biosystems).

### Functional assays

Cell growth was analysed at 24 and 48 hours using the Promega CellTiter Aqueous One Solution cell Proliferation Assay (Promega) according to the manufacturer’s instructions, using Multiskan plate reader (Thermo Labsystems). For this assay cells were plated in triplicate for each transfection condition and time point. The MTS absorbance reading at each time point was normalised to a non-transfected lipofectamine-only control.

Clonogenicity of cells treated with pre-miR-155 or scrambled control was assayed by seeding 400 cells in 1 mL 1% MethoCell MC (in RPMI with 10% FBS per 35 mm plate). Colony number and size were scored microscopically (colony defined as >50 cells) using standard criteria after 14 days at 37°C in 5% CO_2_.

Cell cycle analysis was performed by exposing cells to 1 mL 1% TX-100, 50 μL 50 mg/mL propidium iodide (PI) and 50 μL of 10 mg/mL RNAaseA at 37°C for 30 minutes and analysed by flow cytometry. Analysis of proliferation was conducted by pulsing cells with 10 μM BrdU (Bromodeoxyuridine) for 45 mins, before fixing with absolute ethanol and probing using a primary mouse-anti-BrdU antibody (BD Biosciences) and goat-anti-mouse-APC conjugated secondary antibody (Jackson Laboratories, Pennsylvania, USA). Cells were co-stained with 1 mg/mL PI and treated with 0.5 mg/mL RNAse before flow cytometry analysis. Apoptotic fractions cells were analysed by Annexin V/7-AAD staining using the Annexin V-APC Apoptosis Detection Kit I (BD Pharminogen) according to the manufacturer’s instructions. Intracellular activated caspase-3 was directly measured by staining permeabilised cells (FACSperm, BD Biosciences) with PE Rabbit Anti-Active Caspase-3 (BD Pharmingen). Data collection for these assays was performed using a LSRII flow cytometer (Becton Dickinson, Mountain View, CA), and analysis was performed on FlowJo Software on at least 10,000 acquired events.

### Induction of monocytic differentiation of AML cells

Monocyte differentiation was induced by a 48 hour pulse of 10nM 1,23-dihydroxy-vitaminD3 (Sigma-Aldrich, St Louis, MO). Cells were collected at 0 and 48 hours for total RNA extraction and cell characterization. Cell lineage-specific marker expression was analyzed by flow cytometry in untreated and treated cells at various time points during differentiation. Flow cytometry analysis was performed using CD14-FITC, CD15-PE and CD11b-APC, together with their respective isotype controls (all from BD Pharmigen, San Jose, CA). Cells were incubated with 10uL of a specific antibody or antibody combination and were incubated in the dark for 20 min at room temperature. Cells were washed twice with PBS containing 0.5% bovine serum albumin and 0.1% Sodium Azide and resuspended in 0.5% paraformaldehyde. Cells were analyzed immediately by LSRII flow cytometer with a minimum acquisition of 30,000 events.

### Antibody array methods

Proteome Profiler Human Apoptosis Array Kits from R&D Systems were used to measure levels of apoptosis related proteins according to the manufacturer’s protocol. 200ug of protein was applied per array. In this method, proteins are captured by antibodies spotted on a nitrocellulose membrane, then levels of protein expression are assessed using a HRP-conjugated antibody, followed by chemiluminesce detection. This was analysed using the QuantityOne Software.

## Abbreviations

AML: Acute myeloid leukaemia; NPM1: Nucleophosmin-1; NK: Normal karyotype; LNA: Locked nucleic acid.

## Competing interests

The authors declare no financial or non-financial competing interests.

## Authors’ contributions

CAP carried out the qRT-PCR, apoptosis, cell cycle and cell growth assays, clonogenic, BrdU, differentiation assays, participated in study design and wrote the manuscript. DA carried out apoptosis assays, qRT-PCR and Antibody arrays and helped write the manuscript. TKL carried out qRT-PCR and helped write the manuscript. AB carried out qRT-PCR of patient samples and participated in study design. TTV participated in data interpretation and helped write the manuscript. VY performed the bioinformatic microarray analysis and target prediction. DDM conceived of the study, participated in its design, data interpretation and writing the manuscript. All authors read and approved the final manuscript.

## Supplementary Material

Additional file 1: Table S3 Potential targets of miR-155 predicted by each of MicroCosm, microRNA.org and TargetScan. 72 genes were commonly predicted to be targeted by miR-155 by the three algorithms. Click here for file

Additional file 2: Table S1 Gene Ontology terms enriched in predicted miR-155 targets. Gene ontology analysis was performed using DAVID Functional Annotation Tool, using the permissive 585-gene list which included putative miR-155 targets predicted by two of three bioinformatics databases (TargetScan, microRNA.org, MicroCosm). Click here for file

Additional file 3: Table S2 List of potential miR-155 target genes analysed [24], [25,41,42,44,63-66]. Click here for file

Additional file 4: Figure S1 miR-155 expression levels during MV4-11 cell apoptosis and monocytic differentiation. Expression of miR-155 during **(A)** ARAC induced apoptosis of MV4-11 cells or **(B)** VitD3 induced monocytic differentiation of MV4-11 cells. Data is presented as mean fold change expression of miR-155 + SEM relative to untreated control; RNU6b was used as the reference gene. Paired Two Tailed T-Test did not detect significant differences; n = 3. Click here for file

Additional file 5: Figure S2 Functional effects of miR-155 knockdown in MV4-11 cells. **(A)** VitD3 was used to induce myeloid differentiation in MV4-11 cells transfected with anti-miR155 LNA or CTL. Percentage expression of CD14 + CD11b + cells transfected with anti-miR155 and exposed to VitD3 (+) or PBS (-) for 48 hours did not demonstrate significant difference with miR-155 inhibition **(B)** Transfection of MV4-11 cells with anti-miR155 LNA did not result in a change in the proportion of cells undergoing apoptosis (AnnexinV+). LNA- locked nucleic acid. Statistical significance determined using Paired Two Tailed T-Test; n = 3. Click here for file

Additional file 6: Figure S3 Kaplan-Meier survival analysis demonstrating increased overall survival in patients with miR-155 overexpressing tumours. **(A)** Assessment of microRNA expression from 218 patients with primary or metastatic prostate cancer with a median of 5 years clinical follow-up, demonstrate higher survival probability in patients with higher miR-155 expression, p = 0.0155 [56]**(B)** expression profiling of 38 high-risk ER + breast cancers demonstrate higher OS in patients with high miR-155 expression, p = 0.000121 [55]. Click here for file

Additional file 7: Table S4 List of primer sequences. All mRNA primers were designed to be intron spanning. Click here for file
