# Supplementary material for: MicroRNA-155 as an inducer of apoptosis and cell differentiation in Acute Myeloid Leukaemia
Source: Mol Cancer. 2014 Apr 5;13:79. doi: 10.1186/1476-4598-13-79 (PMC4021368; doi:10.1186/1476-4598-13-79)
Supplement: Additional file 1: Table S3 — Potential targets of miR-155 predicted by each of MicroCosm, microRNA.org and TargetScan. 72 genes were commonly predicted to be targeted by miR-155 by the three algorithms. [file 1476-4598-13-79-S1.doc]

**Additional Table 3: Potential targets of miR-155 predicted by each of MicroCosm, microRNA.org and TargetScan.** 72 genes were commonly predicted to be targeted by miR-155 by the three algorithms.

| ACTR10 | DET1 | LSM14A | SLC12A6 |
| --- | --- | --- | --- |
| ACTA1 | DHX40 | LAMP2 | SPIN3 |
| AICDA | DCLRE1A | MGP | SPI1 |
| AGTRAP | DNAJB7 | MAP3K10 | SKIV2L2 |
| ARRB2 | DYNC1I1 | MBNL3 | SOCS1 |
| BAIAP2L1 | FBXO11 | MYLK | SMARCA4 |
| BCORL1 | FAM105A | MYO10 | SDCBP |
| BOC | FOS | PSIP1 | STXBP5L |
| BRD1 | GNAS | PDE7A | TERF1 |
| CARHSP1 | H3F3A | PHC2 | TPRKB |
| CSNK1G2 | HBP1 | PTPN2 | TAPT1 |
| CEBPB | HIVEP2 | PCDH9 | TRIM32 |
| CDC73 | IKBKE | RCN2 | TP53INP1 |
| CLCN5 | IL13 | RNF123 | USP43 |
| CHD7 | JARID2 | RNF149 | USP8 |
| C8orf4 | KIAA1267 | SALL1 | VPS18 |
| COL21A1 | KIAA1715 | SAP30L | WDR45 |
| CSF1R | LRP1B | SGK3 | WEE1 |
|  |  | SHOX | ZBTB38 |
