# Supplementary material for: MicroRNA-155 as an inducer of apoptosis and cell differentiation in Acute Myeloid Leukaemia
Source: Mol Cancer. 2014 Apr 5;13:79. doi: 10.1186/1476-4598-13-79 (PMC4021368; doi:10.1186/1476-4598-13-79)
Supplement: Additional file 2: Table S1 — Gene Ontology terms enriched in predicted miR-155 targets. Gene ontology analysis was performed using DAVID Functional Annotation Tool, using the permissive 585-gene list which included putative miR-155 targets predicted by two of three bioinformatics databases (TargetScan, microRNA.org, MicroCosm). [file 1476-4598-13-79-S2.doc]

**Additional Table 1: Gene Ontology terms enriched in predicted miR-155 targets.** Gene ontology analysis was performed using DAVID Functional Annotation Tool, using the permissive 585-gene list which included putative miR-155 targets predicted by two of three bioinformatics databases (TargetScan, microRNA.org, MicroCosm).

| **GO** | **GO TERM** | **P value (Bonferroni)** |
| --- | --- | --- |
| GO:0006350 | Transcription | 1.45E-07 |
| GO:0045449 | Regulation of Transcription | 1.37E-07 |
| GO:0006355 | Regulation of Transcription: DNA dependent | 7.02E-05 |
| GO:0051252 | Regulation of RNA metabolic process | 8.94E-05 |
| GO:0006357 | Regulation of transcription from RNA polymerase II promoter | 0.0125 |
| GO:0035295 | Tube Development | 0.0125 |
| GO:0045646 | Regulation of Erythrocyte differentiation | 0.0119 |
| GO:0048598 | Embryonic morphogenesis | 0.0123 |
| GO:0045637 | Regulation of Myeloid Cell Differentiation | 0.0118 |
| GO:0045944 | Positive regulation of transcription from RNA pol II promoter | 0.0169 |
| GO:0045893 | Positive regulation of transcription, DNA dependent | 0.0277 |
| GO:0051254 | Positive Regulation of RNA metabolic process | 0.030 |
| GO:0006915 | Apoptosis | 0.0324 |
| GO:0012501 | Programmed cell death | 0.0414 |
| GO:0048568 | Embryonic organ development | 0.0431 |
| GO:0016568 | Chromatin modification | 0.0423 |
| GO:0010628 | Positive regulation of gene expression | 0.0475 |
