# Supplementary material for: MicroRNA-155 as an inducer of apoptosis and cell differentiation in Acute Myeloid Leukaemia
Source: Mol Cancer. 2014 Apr 5;13:79. doi: 10.1186/1476-4598-13-79 (PMC4021368; doi:10.1186/1476-4598-13-79)
Supplement: Additional file 3: Table S2 — List of potential miR-155 target genes analysed [24], [25,41,42,44,63-66]. [file 1476-4598-13-79-S3.doc]

**Additional Table 2: List of potential miR-155 target genes analysed.**

| **Gene** | **Function** |
| --- | --- |
| *ARNTL* | Beta subunit of HIF1 transcription factor; *ARNTL* is repressed by miR-155 ; newly linked to cancer through its regulation of the circadian rhythm, p53 activation and its dysregulation is thought to be anti-apoptotic. |
| *BCAT1 (CTNNB1)* | Encodes for β-catenin protein, an integral part of the Wnt signaling cascade; *CTNNB1*, inhibitor to *GSK3b*, and its receptor Frizzled are targets of miR-155 . |
| *CEBPα* | Arrests cell proliferation through inhibition of *CDK2* and *CDK4*. Implicated in AML and normal haematopoiesis. |
| *CEBPβ* | Regulates genes important in immune and inflammatory responses; critical for normal macrophage function. Implicated in ALL. |
| *c-JUN* | In combination with c-FOS, forms the activator protein 1 (AP-1) transcription factor which controls proliferation, differentiation and apoptosis . |
| *CXCR4* | An alpha-chemokine receptor specific for its ligand SDF-1, it is important in directing homing of HSCs into bone marrow after transplant; miR-155 is predicted to repress *CXCR4* . |
| *CCND1* | Forms a complex with and functions as a regulatory subunit of CDK4 and CDK6 kinases, whose activity is required for G1/S cell cycling. |
| *EPAS1 (HIF2a)* | Contains a basic helix-loop-helix dimerisation domain and a domain important in signal transduction pathways which respond to hypoxia; recently EPAS1 has been linked to cancer development . |
| *FOS* | Dimerises with c-JUN to form AP-1 transcription factor . A target of miR-155 based on bioinformatics. |
| *FOXO3* | Triggers apoptosis through upregulation of genes Bim and PUMA. A target of miR-155 based on bioinformatics. |
| *GFI1* | A repressor of genes in lymphoid and myeloid differentiation, controls T-cell receptor signalling, regulates endotoxin-mediated TLR inflammatory response and cell cycle progression; GFI11 is targeted by miR-155 . |
| *HIF1α* | Regulates cellular/systemic responses to hypoxia; Involved in myeloid differentiation ; Knocking out HIF1α kills leukaemic stem cells ; miR-155 targets HIF1α in myeloproliferative disorders . |
| *HOXA9* | Encodes for Homeobox protein Hox-A9; Fusion with NUP98 gene has been linked to myeloid leukemogenesis. Interacts with MEIS1 and PBX1 . |
| *JAK2* | Involved in signaling with type II cytokine receptor family, GM-CSF receptor family, gp130 receptor family and other single chain receptors Epo-R and Tpo-R; Suggested role in CBF-AML; A direct target of miR-155 based on bioinformatics and previous studies . |
| *JARID2* | Codes for a DNA-binding protein that functions as a transcriptional repressor. JARID2 interacts with the Polycomb repressive complex 2 (PRC2) that plays an essential role in regulating gene expression. A target of miR-155 based on bioinformatics. |
| *MEIS1* | Encodes for Homeobox protein Meis1, a protein from the TALE family of homeodomain-containing proteins; Interacts with HOXA9 and PBX1 ; A target of miR-155 based on bioinformatics. |
| *MPO* | Myeloperoxidase is found in granules of neutrophils. Since miR-155 predicted targeting of critical genes (PU.1, FOS and MEIS1) involved in myeloid differentiation , MPO levels may be affected by altering miR-155 levels. |
| *MYC* | Binds on Enhancer Box sequences and recruits histone acetyltransferases to regulate global chromatin structure; Downregulation of MYC can lead to apoptosis; miR-155 has been predicted to repress *MYC* . |
| *PBX1* | Encodes for Pre-B-cell leukemia transcription factor 2, a protein from the TALE/PBX homeobox family; Binds to TLX1 promoter to activate its transcription and is involved in t(1;19) translocation in acute pre-B-cell leukaemias. Interacts with HOXA9 and MEIS1 . |
| *TRIB2* | Regulates activation of MAPK kinases; Interacts with HOXA9 in AML. |
