# Supplementary figures and images for: MicroRNA-155 as an inducer of apoptosis and cell differentiation in Acute Myeloid Leukaemia
Source: Mol Cancer. 2014 Apr 5;13:79. doi: 10.1186/1476-4598-13-79 (PMC4021368; doi:10.1186/1476-4598-13-79)

## Slide 1
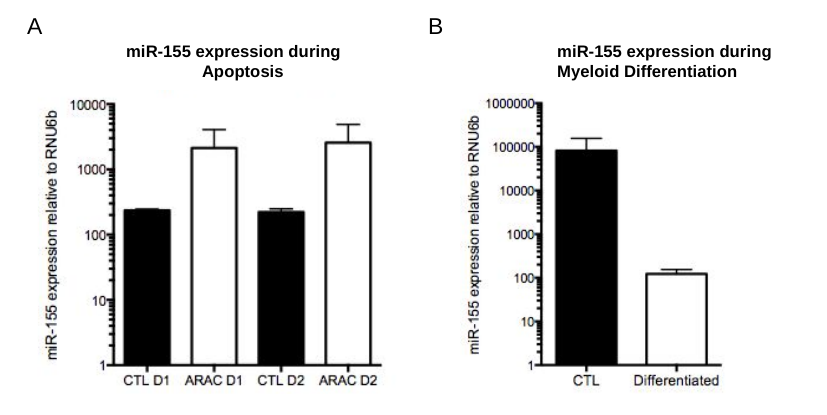

A
B
miR-155 expression during
 Apoptosis
miR-155 expression during Myeloid Differentiation

Supplement: Additional file 4: Figure S1 — miR-155 expression levels during MV4-11 cell apoptosis and monocytic differentiation. Expression of miR-155 during (A) ARAC induced apoptosis of MV4-11 cells or (B) VitD3 induced monocytic differentiation of MV4-11 cells. Data is presented as mean fold change expression of miR-155 + SEM relative to untreated control; RNU6b was used as the reference gene. Paired Two Tailed T-Test did not detect significant differences; n = 3. [file 1476-4598-13-79-S4.pptx]

## Slide 1
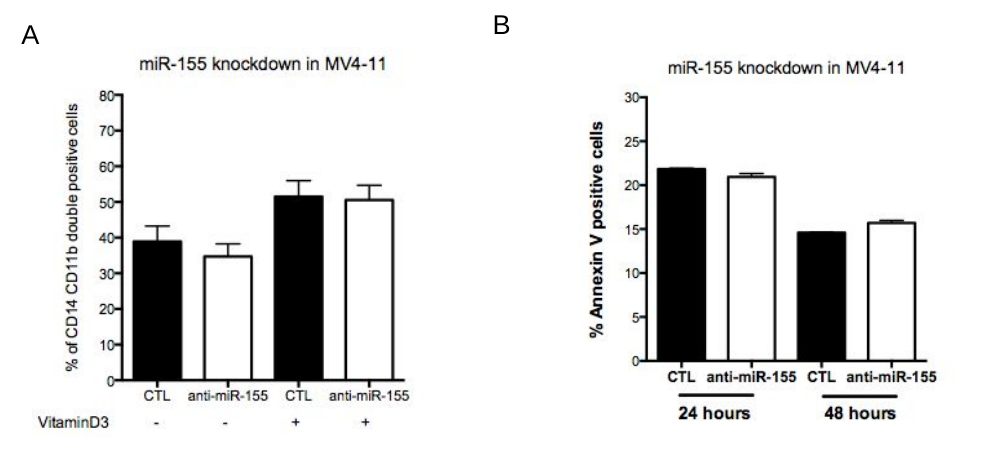

B
A

Supplement: Additional file 5: Figure S2 — Functional effects of miR-155 knockdown in MV4-11 cells. (A) VitD3 was used to induce myeloid differentiation in MV4-11 cells transfected with anti-miR155 LNA or CTL. Percentage expression of CD14 + CD11b + cells transfected with anti-miR155 and exposed to VitD3 (+) or PBS (-) for 48 hours did not demonstrate significant difference with miR-155 inhibition (B) Transfection of MV4-11 cells with anti-miR155 LNA did not result in a change in the proportion of cells undergoing apoptosis (AnnexinV+). LNA- locked nucleic acid. Statistical significance determined using Paired Two Tailed T-Test; n = 3. [file 1476-4598-13-79-S5.pptx]

## Slide 1
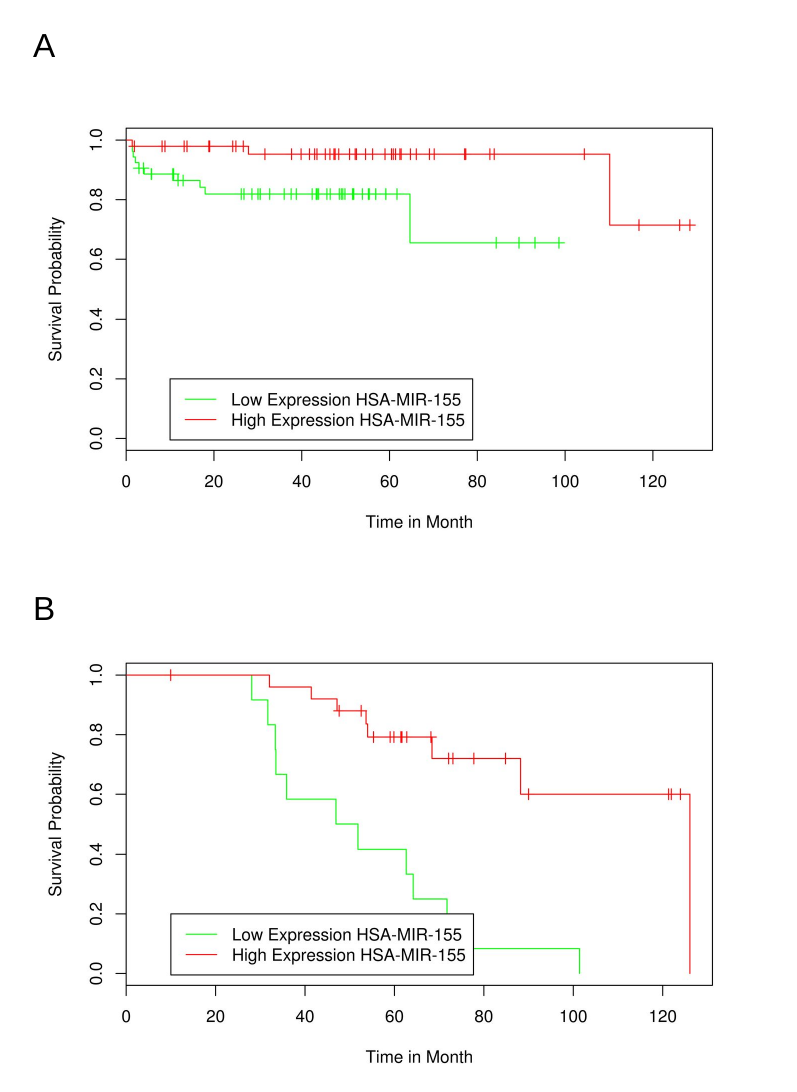

A
B

Supplement: Additional file 6: Figure S3 — Kaplan-Meier survival analysis demonstrating increased overall survival in patients with miR-155 overexpressing tumours. (A) Assessment of microRNA expression from 218 patients with primary or metastatic prostate cancer with a median of 5 years clinical follow-up, demonstrate higher survival probability in patients with higher miR-155 expression, p = 0.0155 [56](B) expression profiling of 38 high-risk ER + breast cancers demonstrate higher OS in patients with high miR-155 expression, p = 0.000121 [55]. [file 1476-4598-13-79-S6.pptx]
