# Supplementary material for: MicroRNA-155 as an inducer of apoptosis and cell differentiation in Acute Myeloid Leukaemia
Source: Mol Cancer. 2014 Apr 5;13:79. doi: 10.1186/1476-4598-13-79 (PMC4021368; doi:10.1186/1476-4598-13-79)
Supplement: Additional file 7: Table S4 — List of primer sequences. All mRNA primers were designed to be intron spanning. [file 1476-4598-13-79-S7.doc]

**Additional Table 4: List of primer sequences.** All mRNA primers were designed to be intron spanning.

| **Gene** | **Gene Name** |  | **Primer Sequences (5’ to 3’)** |
| --- | --- | --- | --- |
| *miR-155* | Hsa-miR-155 | F | TTAATGCTAATCGTGATAGGGGT |
| *RNU6B* | RNA, U6B small nuclear | F | CGCAAGGATGACACGCAAATTCGT |
| *RNU43* | RNA, U48 small nuclear | F | AACTCTGAGTTGTCGCTGATC |
| *ARNTL* | Aryl Hydrocarbon receptor nuclear translocator-like | F | GCACGCGATAGATGGAAAGT |
|  |  | R | TTGCCTATGACATTCTGCAAG |
| *β-ACTIN (ACTB)* | Actin, beta | F | TTCTACAATGAGCTGCGTGTG |
|  |  | R | GGGGTGTTGAAGGTCTCAAA |
| *CCND1* | Cyclin D1 | F | CCCTCGGTGTCCTACTTCAA |
|  |  | R | CTCCTCGCACTTCTGTTCCT |
| *CEBPA* | CCAAT/enhancer binding protein (C/EBP), alpha (CEBPA) | F | CTTGTGCCTTGGAAATGCAA |
|  |  | R | GCTGTAGCCTCGGGAAGGA |
| *CEBPB* | CCAAT/enhancer binding protein (C/EBP), beta (CEBPB) | F | AACCAACCGCACATGCAGAT |
|  |  | R | GGCAGAGGGAGAAGCAGAGAGT |
| *JARID2* | Jumonji, AT rich interactive domain 2 | F | AGCAGGCTTCAGCTAACCAC |
|  |  | R | TACACCTGCACCCAGAGATG |
| *c-JUN* | Jun proto-oncogene | F | AGGAGGAGCCTCAGACAGTG |
|  |  | R | AGCTTCCTTTTTCGGCACTT |
| *CTNNB1 (BCAT1)* | Catenin (cadherin-associated protein), beta 1, 88kDa | F | CAGAAAATGGTTGCCTTGCT |
|  |  | R | TCAGCACTCTGCTTGTGGTC |
| *CXCR4* | Chemokine (C-X-C motif) receptor 4 | F | GAAGCTGTTGGCTGAAAAGG |
|  |  | R | CTCACTGACGTTGGCAAAGA |
| *EPAS1 (HIF2A)* | Endothelial PAS domain protein 1 | F | CAACCTCAAGTCAGCCACCT |
|  |  | R | TGCTGGATTGGTTCACACAT |
| *FOS* | Fos proto-oncogene | F | CCGGGGATAGCCTCTCTTAC |
|  |  | R | GTGACCGTGGGAATGAAGTT |
| *FOXO3* | Forkhead Box O3 | F | CTTCAAGGATAAGGGCGACA |
|  |  | R | AGTTCCCTCATTCTGGACCC |
| *GFI11* | Growth Factor Independent Transcription Repressor 1 | F | AGCCTGGAGCAGCACAAA |
|  |  | R | TGGATAAGCAGGTGTGTGGA |
| *HIF1A* | Hypoxia inducible factor 1, alpha subunit | F | TCATCCAAGAAGCCCTAACG |
|  |  | R | TCCATTTTTCGCTTTCTCTGA |
| *HOXA9* | Homeobox A9 | F | CAATAACCCAGCAGCCAACT |
|  |  | R | CAGTTCCAGGGTCTGGTGTT |
